# A Randomized, Double-Blind, Controlled Trial Protocol for Therapeutic Neuroscience Education in Chronic Migraine Patients: A Clinical–Neurophysiological Combined Study Design

**DOI:** 10.3390/mps8020022

**Published:** 2025-02-20

**Authors:** Matteo Castaldo, Tiziana Atzori, Angela Comanducci, Giacomo Querzola, Chiara-Camilla Derchi, Daniele Lovattini, Carlo Manzoni, Carlo Lovati, Francesca Baglio, Paola Tiberio, Rita De Sanctis, Simone Sarasso, Alessandro Viganò

**Affiliations:** 1 IRCCS—Fondazione Don Carlo Gnocchi, 20148 Milan, Italy; matteo.castaldo@unipr.it (M.C.); tatzori@dongnocchi.it (T.A.); acomanducci@dongnocchi.it (A.C.); cderchi@dongnocchi.it (C.-C.D.); fbaglio@dongnocchi.it (F.B.); avigano@dongnocchi.it (A.V.); 2 Headache Center, Luigi Sacco University Hospital, 20157 Milan, Italy; giacomo.querzola@asst-santipaolocarlo.it (G.Q.); carlo.lovati@tiscali.it (C.L.); 3 Department of Biomedical and Clinical Sciences, University of Milan, 20122 Milan, Italy; danielelovattini@gmail.com (D.L.); carlo.manzoni@studenti.unimi.it (C.M.); simone.sarasso@unimi.it (S.S.); 4 Department of Biomedical Sciences, Humanitas University, 20072 Pieve Emanuele, Italy; paola.tiberio@cancercenter.humanitas.it; 5 Medical Oncology and Hematology Unit, IRCCS Humanitas Research Hospital, 20089 Rozzano, Italy

**Keywords:** disability, rehabilitative approach, RCT, education, IDAP, blink reflex, predictive factor, pain neuroscience education

## Abstract

Chronic migraine (CM) is a highly disabling condition, affecting about 2% of the global population. Non-pharmacological treatments can be optimal for their non-invasive nature. This prospective, randomized, double-blind, controlled trial aimed to test the efficacy of therapeutic neuroscience education (TNE) in CM. Early response biomarkers were also evaluated. A total of 80 CM patients were consecutively enrolled and randomly allocated to TNE or a general education program. Treatment effectiveness was evaluated at baseline (T1) and 2 months after the end of treatment (T4). We collected the responses to disability and comorbidity questionnaires at the start (T1) and end of treatment (T3, 10 weeks after start). Early response biomarkers were evaluated at screening (T0) and mid-way through the process (T2, 5 weeks after start). We expected that TNE would provide a greater benefit than the general education program, which served as the primary outcome of this study. We also expected that a change in clinical and neurophysiological measures could potentially occur, reflecting plasticity-induced reorganization and predicting clinical response. This is the first study selectively exploring the effect of TNE as a standalone treatment for CM. A new, effective treatment regime without interactions with other medication could be of great interest as an addition to migraine therapeutic strategies.

## 1. Introduction

Migraine represents the seventh most common cause of disability in the global population and the first cause of disability in adults under 50 years of age [1]. Compared to the episodic form, chronic migraine (CM), i.e., >15 days/month, entails a greater disability, lower individual productivity, worse quality of life, and larger expenses (an average of EUR 2250 ± 1796 per person per year) [2,3]. CM affects 2% of the global population, and half of patients with headache are referred to specialized headache centers [4,5]. About 3% of episodic migraineurs convert to CM every year [6], with the most important risk factors being obesity, depression, stressful life events, and overuse of symptomatic medication, which constitutes medication-overuse headache (MOH) [4].

To date, pharmacological treatments represent the mainstay of the therapeutic management of CM, with non-pharmacological (e.g., neuromodulatory [7], rehabilitative [8], or psychotherapeutic [9,10]) approaches remaining less frequently utilized because they are supported by a lower level of evidence [11].

However, the success rate of preventive pharmacological therapies is lower than 50% for CM [12] because of drug inefficacy, frequent side effects, comorbidities, or drug-to-drug interactions [12,13,14]. Monoclonal antibodies, released on the market in recent years, are very effective but limited in prescription by their high cost, either for private or public health systems.

Interestingly, while comorbidities generally represent a drawback for the pharmacological management of CM, this is not always true for some non-pharmacological approaches, such as neuromodulation and psychotherapy, where some comorbidities are positively correlated with clinical benefits [15], suggesting that non-pharmacological options can have a role in treating patients with complications.

Therapeutic neuroscience education (TNE) is a non-pharmacological approach with some level of evidence for migraine management, although the exact parameters (e.g., delivery methods, number of sessions) remain to be elucidated [16,17,18].

TNE is defined as an “educational program aimed at improving the comprehension of pain mechanism, enhancing the coping strategies and increase the patients’ quality of life” [19]. In contrast, conventional anatomical and biomedical education not only showed limited or no efficacy in decreasing pain and disability in patients but might even increase fear, catastrophizing, and pain levels, inducing a nocebo effect [20]. TNE aims to increase knowledge of pain neurophysiology and neurobiology, pain representation, and the meaning of pain and to improve coping strategies [21,22,23]. It also shows effectiveness in reducing pain intensity and migraine disability and improving patients’ moods, with a positive clinical benefit in terms of pain rating, pain-related disability, catastrophizing, and physical performance [24,25] when applied to patients with chronic musculoskeletal pain.

Functional magnetic resonance studies showed that TNE exerts an effect by reducing the activity of several areas belonging to the cerebral pain network [21], suggesting that this intervention could be monitored by neural biomarkers.

Migraine prevention generally requires several months of treatment, either with pharmacological or non-pharmacological therapies. Therefore, a predictive marker could be of great interest, since an early change occurring before the effect of treatment could help in selecting patients or reducing the time of administration of the treatment itself if unnecessary.

Several available and easy-to-perform neurophysiology techniques can be used to highlight the benefit of systemic [26], local [27], and neuromodulatory [7] treatments for migraine. The intensity dependence of auditory-evoked potentials (IDAP), a non-invasive measurement of serotonergic firing from brainstem to cortex, predicted recovery from chronic to episodic migraine after the administration of a single greater occipital nerve block [27].

Also, the nociceptive blink reflex (nBR) was found to be sensitive to changes in excitability induced by transcranial magnetic stimulation and flashlight, biofeedback, and some medications [28,29,30].

Clinically, quantitative sensory testing (QST) was found to effectively assess somatosensory function and differentiate between various phases of the migraine cycle, as responses to different stimuli vary throughout these phases [31].

The primary objective of this study is to evaluate the efficacy of TNE as a preventive treatment for CM compared to a general education protocol, with a focus on reducing monthly headache days from baseline to follow-up. Additionally, we aim to identify potential predictive factors through neurophysiological assessments.

The primary outcome is the reduction in migraine days, as the observed improvement should be at least comparable to that achieved with other therapies. Accurately predicting TNE effectiveness at baseline or early in the treatment process will help identify patients with a high likelihood of response, ensuring they receive appropriate long-term, high-commitment care.

We hypothesize that TNE will reduce monthly headache days and that mid-treatment changes in IDAP, nBR, and QST assessments will precede clinical improvement, serving as potential biomarkers.

## 2. Materials and Methods

### 2.1. Study Cohort

Consecutive CM patients will be recruited for the study. Eligibility criteria will include a CM diagnosis (with or without MOH) based on the International Classification of Headache Disorders (ICHD-3) criteria [32] and age > 18 years, as recent data suggest TNE is effective in older adults as well [33]. Patients will be enrolled consecutively. Exclusion criteria will include an ICHD-3 diagnosis of another primary or secondary headache disorder (except for MOH), neurological or psychiatric conditions that could affect compliance or neurophysiological assessments (e.g., neurodegenerative disorders, multiple sclerosis, epilepsy, schizophrenia, severe anxiety/depression with HADS >15), chronic pain conditions (e.g., low-back pain, chronic pelvic pain), severe comorbidities related to migraine or systemic diseases requiring concurrent medical treatment, and malignancy. Patients on migraine preventive therapy will be included if their prophylaxis has remained unchanged and ineffective for at least three months prior to recruitment. This treatment will not be down-tapered or discontinued at the study’s onset. Relevant demographic data will also be collected.

Migraine burden will be assessed throughout the study using a headache diary (tracking headache days, pain intensity, and acute medication use) and several validated questionnaires, including the Migraine Disability Assessment (MIDAS), the Headache Impact Test (HIT-6), the Central Sensitization Inventory (CSI), the Pain Catastrophizing Scale (PCS), and the Hospital Anxiety and Depression Scale (HADS) [34,35,36,37,38].

### 2.2. Outcome Measures

The primary aim of the present study is to test the efficacy of TNE as a preventive treatment for CM compared to a general education protocol. The primary outcome will be the reduction in monthly headache days from baseline to follow-up. Secondary endpoints will include (i) the interruption rate of MOH; (ii) the proportion of patients achieving a >50% reduction in headache days (i.e., responders) at follow-up; (iii) differences in biomarkers (IDAP, nBR, and the QST) between responders and non-responders, along with the correlation between these biomarker values and clinical improvement in headache day reduction to assess their potential as efficacy predictors; and (iv) significant reductions in disability and migraine burden scores.

### 2.3. Randomization, Schedule, and Adherence

Patients will be randomly assigned at a 1:1 ratio using a pre-generated randomization list created in SPSS by an experimenter who will not be involved in patient recruitment, treatment, or data analysis. Participants will be required to attend a full course of 10 lessons, with the first and last sessions conducted in person and the remaining eight delivered remotely via PowerPoint presentations. Lessons will be held in small groups, with the real and control education groups scheduled on different days to prevent interaction between participants from different study arms. The treatment will be considered valid if patients attend at least 8 out of 10 lessons. In case of absence, the PowerPoint presentation from the missed session will be sent to the participant. Additionally, a brief Q&A session will be conducted before the next lesson to review the content and address any questions.

### 2.4. Study Design

After screening for inclusion/exclusion criteria (T0), patients will complete a headache diary for one month before entering the study (T1). At this point, they will undergo QST, neurophysiological assessments, and questionnaire evaluations before being randomly assigned to either the experimental (TNE-A) or control (CON-A) group (further details provided in the next section).

**Figure 1:**
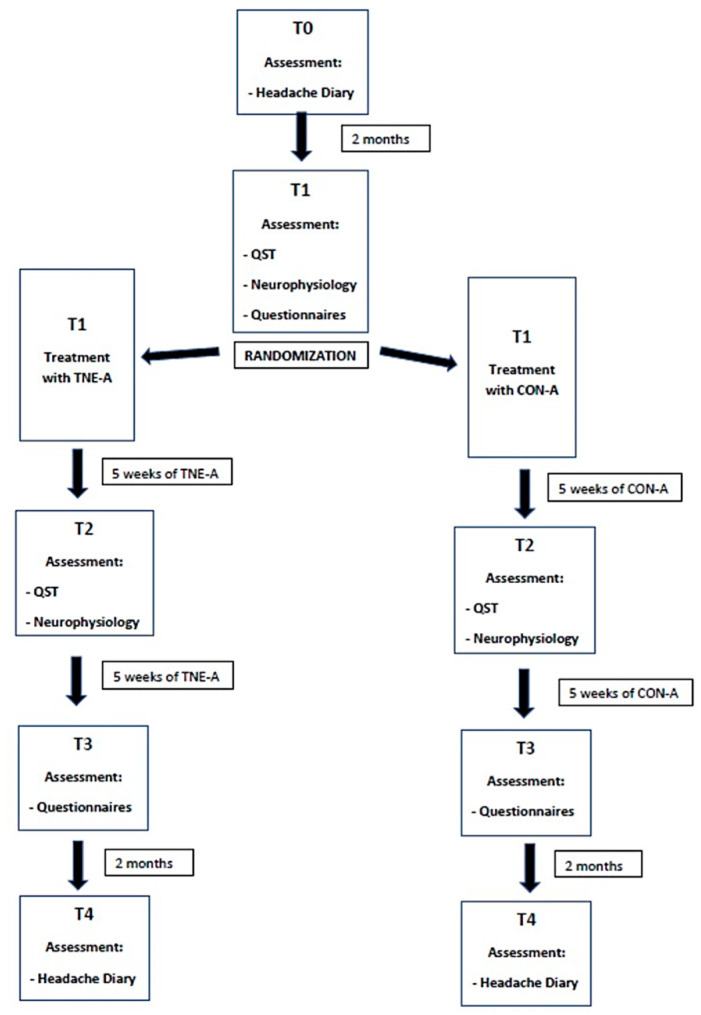
Timeline of the study design. QST, quantitative sensory testing; TNE-A, therapeutic neuroscience education arm; CON-A, control arm.

Following the 5th lecture (T2), patients will repeat the QST and neurophysiological assessment (T2). Headache frequency and questionnaire data will be collected again at the end of the 10-session program (T3). Additionally, headache burden will be reassessed two months after completing the TNE program (T4) (see Figure 1). This protocol follows the Standard Protocol Items: Recommendations for Interventional Trials (SPIRIT) guidelines [39] and is designed and will be reported in accordance with the Consolidated Standards of Reporting Trials (CONSORT) [40].

### 2.5. Quantitative Sensory Testing (QST)

For quantitative sensory testing, the pressure pain threshold (PPT) will be measured with an electronic algometer (Somedic AB, Sösdala, Sweden). The probe area will be 1cm^2^, and the force will increase at a rate of approximately 30 kPa/s. Patients will be seated comfortably for hand and leg measurements or lying on their non-tested side for temporalis muscle assessments. They will be instructed to press a stop button as soon as they perceive a change from pressure to pain. To familiarize patients with the procedure, a training test will be conducted on a neutral body part before measurements begin. PPT will be measured three times at each assessment site, and the average value will be used for analysis.

PPTs will be assessed in the trigeminal area over the anterior column of the temporalis muscle. Since previous studies showed no side-to-side differences in PPT over the trigeminal area in migraine patients with unilateral migraine [41], measurements will be taken on the symptomatic side for unilateral migraine cases and on the dominant side for patients with bilateral or shifting-side migraines.

PPTs will be also assessed in the extra-trigeminal area over the second metacarpophalangeal joint of the dominant hand and over the tibialis anterior muscle of the dominant side to evaluate widespread pressure pain hypersensitivity. These sites are commonly used to assess widespread hyperalgesia as a sign of central sensitization in chronic pain conditions [42]. Studies have shown that migraine patients exhibit lower PPTs in the trigeminal, cervical, second metacarpal, and tibialis anterior regions compared to healthy individuals [43]. However, a recent systematic review suggested that migraine patients have lowered PPTs in local areas but not in non-local areas [44].

Wind-up refers to the progressive increase in the excitability of trigeminal and spinal dorsal horn wide dynamic range neurons (WDRs) evoked by repetitive identical (same frequency) stimulation of primary afferent nociceptive C-fibers (homosynaptic potentiation) [45]. The human equivalent of this phenomenon is temporal summation, which manifests as an increased pain perception with repetitive, constant nociceptive stimulation. To assess temporal summation, the wind-up ratio (WUR) will be calculated. Mechanical pin-prick-evoked pain will be tested using a 50.1 g device (Aalborg University, Aalborg, Denmark) over the anterior column of the temporalis muscle. The symptomatic side will be assessed for unilateral migraine patients, while the dominant side will be used for bilateral or shifting-side migraine cases. Patients will lie on their non-tested side and rate their pain on a 0–10 scale for both the first and last of 10 identical stimuli applied at the same location on the temporalis muscle at a frequency of 1 stimulus per second. The difference between the last and first pain ratings will be calculated, with a positive WUR indicating increased temporal summation [46].

### 2.6. Nociceptive Blink Reflex (nBR)

The nBR will be recorded using a previously established setup for migraine research [28]. Electrical stimulation will be applied via an electrode placed near the right supraorbital foramen. Two pairs of recording electrodes will be placed bilaterally over the orbicular oculi muscle: the active electrode over the inferior portion and the reference electrode on the lateral part. A ground electrode will be placed on the nose. Recordings will be conducted at a sampling rate of 5000 Hz and a sweep duration of 150 ms. A 0.2 ms square wave with an ascending-descending sequence with 0.2 mA of intensity steps will be used to probe stimulation. Both the perception threshold (the intensity at which the patient detects at least 50% of the stimuli) and pain threshold (the intensity at which the patient reports pain in at least 50% of the stimulations) will be determined for each subject. During the recording, stimuli will be delivered at 1.5 times the pain threshold to minimize habituation over time. The inter-stimulus interval will be pseudo-randomized between 15 and 17 s. Ten rectified EMG responses will be recorded and averaged offline, with the first response of each nBR discarded to eliminate startle contamination. The amplitude of the R2 reflex response will be quantified as the value of the area under the curve (AUC). To minimize variability, the AUC will be normalized for stimulation intensity (according to the formula AUC/i^2^), as recommended [47].

### 2.7. Intensity Dependence of Auditory Evoked Potentials (IDAP)

IDAP recordings will be conducted following established protocols [27,48,49]. Auditory evoked potentials will be delivered at four different intensities (60, 70, 80, 90 dB) in a pseudo-randomized order. For each intensity, 90 stimuli will be delivered. The sampling rate will be set at 4000 Hz and sweep duration at 400 ms (including 50 ms before and 350 ms after stimulus onset). Traces will be filtered offline with a 1–20 Hz pass-band filter. N1 will be identified as the most negative deflection occurring between 60–150 ms, while P2 will be defined as the most positive deflection between 120–200 ms. After removing electroencephalographic traces contaminated by artifacts, N1P2 peak-to-peak amplitudes will be measured and averaged for each intensity level. The IDAP slope will be modeled as a linear function interpolating the N1P2 amplitudes across the four intensity levels, with the slope value (μV/10 dB) representing the degree of IDAP.

Since this study involves patients with CM, neurophysiological and clinical evaluations will not be conducted during a strictly “interictal” period. Instead, assessments will take place at least 48 h after a migraine attack (defined as an episode with pain intensity >7/10, nausea, or marked photophobia).

Patients will be contacted two days post-assessment to verify whether any migraine attacks occurred within that period. Additionally, patients must refrain from using analgesics, triptans, or other rescue medications for at least 24 h before the assessment.

Recordings will be performed using a Natus Nicolet Edx system (Middleton, WI, USA).

### 2.8. Therapeutic Neuroscience Education

QST provides insight into the overall state of central nervous system (CNS) sensory amplification. It is typically conducted by applying peripheral stimuli (e.g., electrical, mechanical, thermal, or vibratory) and evaluating the CNS response. For this reason, QST is considered an indirect measure of sensory processing [50].

Studies have demonstrated that QST can predict chronic pain trajectories, assess the risk of pain chronification, and determine treatment outcomes [51].

Different QST modalities have been extensively investigated in migraine patients since the 1950s [34], yet significant heterogeneity remains in assessment methods. A recent systematic review with meta-analysis on QST in migraine patients concluded that alterations in somatosensory function in migraine are specific to the stimulus modality, measurement approach, and anatomical location [52].

The content of the program in the TNE-A group will align with established literature on the topic adapted from “Explain Pain” and “Explain Pain Supercharged” [53,54]. The TNE intervention will consist of ten one-hour group lectures delivered over a 10-week period. Groups will be composed of a moderate number of participants (4 to 8 individuals) to encourage interaction and participant feedback. The program will be administered by two dedicated experts, both of whom underwent standardized training by the same specialist to ensure consistency and reliability in the intervention, as supported by findings from an unpublished pilot feasibility study.

The TNE intervention will be delivered as follows. Initially, we will introduce a simple definition of pain according to the International Association for the Study of Pain and provide an overview of neuron functions, neuroanatomy, and pain classification (e.g., neuropathic, nociceptive, nociplastic). We will then discuss the origins of pain, addressing common misconceptions (e.g., nociception equals pain, pain intensity directly correlates with injury) and challenge migraine-specific misunderstandings (e.g., the belief that effective treatment must eliminate pain rather than promote significant clinical improvement). This will aim to facilitate a reconceptualization of pain for the patients. Next, we will explore pain modulation and the existence of descending/ascending and facilitator/inhibitory pathways underlying the role of our “internal pharmacy”. Patients will learn about the influence of feelings, thoughts, emotions, and beliefs on pain perception. Practical examples, scientific papers, and self-analysis will be used to demonstrate how various factors can impact pain experience. Metaphors such as “the brain as a protectometer” and “the brain as a spam filter” will be introduced to clarify these concepts. We will then explain what happens when pain becomes chronic and the key elements that facilitate chronicity. We will explain the concepts of central sensitization and brain plasticity, which are very important concepts in chronic pain patients. It is crucial for patients to understand that “the brain is trainable, and pain chronicity can be modulated by the brain” and that “we can teach our brain to re-activate its protective mechanisms” to foster clinical improvement. The later sessions will focus on how context and communication influence pain experience, particularly the placebo and nocebo effects and the role of positive language (“good words”). Stress management techniques will also be covered, considering that stress can trigger migraine attacks. Helping patients manage stress effectively will enable them to focus on the present moment, become aware of their thoughts, and accept them without judgment. The final session will recap the key points discussed throughout the previous nine sessions and emphasize the importance of lifestyle modifications and long-term management strategies for migraine control.

The program in the CON-A group will also consist of ten learning sessions, but instead of focusing on the individual–pain interaction, it will cover the following topics: the classification of headaches according to the ICHD-3 criteria; an overview of migraine, including its definition, symptoms, epidemiology, and genetics; migraine pathophysiology, where we will explain both mechanisms leading to pain during an attack and those that contribute to the recurrence of migraine attacks over time; a biomedical model of pain (explaining the association between tissue damage/inflammation and pain perception); the definition of chronic migraine, the mechanisms of migraine chronification, and how migraines can revert to an episodic form; an explanation of the prodromal and postdromal phases of migraine as well as trigger factors; a discussion on the classes of medications used in migraine management and general information on treatment strategies; the role of passive interventions in pain management; and a look at migraine comorbidities as well as the anatomy and biomechanics of the cervical spine. The final session will be a summary of the key points discussed in the previous nine sessions.

In both groups, patients will be assigned homework between sessions, primarily consisting of cognitive exercises such as reflecting on the material covered or explaining the concepts they learned to someone else in simple terms. This approach will help patients mentally organize the new information, reinforcing their understanding and retention of these concepts.

### 2.9. Blinding Procedure

To protect the blinding, patients will be informed that they will be assigned to one of two groups for an educational therapeutic treatment, without further details about the differences between the groups. A brief overview of both the TNE and the control content will be provided to patients without indicating which group they belong to. Patients will be told that the focus of the sessions will vary depending on the group they are in. Additionally, the lessons will be conducted in small groups, with patients from different arms scheduled on separate days and times, both in-person and remotely, to ensure that patients in one group only interact with others from the same group.

While the TNE expert will not be blinded to the intervention, the evaluation team (including the neurologist, QST expert, and neurophysiologist) as well as the data analyst will remain blinded to the patients’ group assignments to uphold the double-blind design. After the final (tenth) lesson, each patient will be asked to guess which group they believe they were assigned to in order to assess the effectiveness of the blinding process.

### 2.10. Plans for Analysis

An a priori calculation of the sample size was performed based on the primary endpoint. In line with studies on CM with MOH, we aim for a between group difference of 3.0 monthly headache days at T4 and a standard deviation of 4.5 [55]. Using an alpha error of 5%, a power of 80%, and a medium effect size, the calculated sample size is 70 patients. Considering a reasonable dropout rate, we plan to recruit 80 patients.

To assess the treatment’s effects on clinical and instrumental outcomes, a generalized linear mixed model for repeated measures (*p*-value < 0.05) will be performed on the primary outcome and, in a sub-analysis, on secondary outcomes. The model will include group and time as fixed effects, time by group as an interaction term, and the outcome as the dependent variable. Covariates will be selected from those identified as significantly different between groups [10]. A correlation analysis will also be conducted between migraine disability indexes and clinical/neurophysiological measures to identify potential biomarkers. False discovery rate correction will be applied to reduce type-I errors.

The number of adverse events (AEs) during the treatment period will be counted separately for the TNE-A and CON-A groups and classified according to common terminology criteria for adverse events (CTCAEs). An AE is defined as any adverse medical event associated with the use of a treatment in humans, regardless of whether it is considered related to the treatment. Causality (relationship to study treatment), action taken, and outcome will be summarized separately.

## 3. Discussion

This will be the first randomized clinical trial testing the efficacy of neuroscience therapeutic education as a standalone treatment for CM patients. While such treatments hold significant potential, their development faces several challenges, such as creating an appropriate sham. Instead of a traditional sham group, we opted to design a custom control group, delivering migraine-related information in a structured format. This approach aims to create a control group that is both plausible and distinct from the active group, which is critical since patients need to remain unaware of their treatment allocation over the 2.5-month duration. However, to avoid diluting the effect of the active treatment, we ensured the control arm content did not overlap with the educational material in the active arm. Therefore, in CON-A, we chose to provide science-based information about migraine, covering aspects like epidemiology, pathophysiology, and basic clinical knowledge, with a relatively high technical detail level. The content was based on a popular migraine educational book [56]. However, to maintain the distinction, we intentionally excluded content that could influence the patients’ understanding of pain or improve their coping strategies. The goal was to keep the education on “what migraine pain is” (in the control group) separate from “what migraine pain means” (in the active group).

Adherence is another critical consideration. Given the duration of the treatment, with several lessons over an extended period, there is a potential risk of high dropout rates. However, our protocol has been designed to make attendance manageable, with only one lesson per week. Eight out of ten lessons can be attended remotely through a conference platform and last about an hour. In three instances, patients are required to admit to the hospital: at T1 to perform baseline assessment and receive the first lesson in presence, midway thought the TNE treatment to collect the second neurophysiology assessment, and at the end for the recap lesson which has the scope of performing a recap of the prior lessons and reinforce the learning. Although attendance is required, it is not as frequent as other protocols, such as neuromodulation treatments, which typically require visits two to three times a week for one to two months. Patients who decide to discontinue the study will be referred to alternative treatments and will be classified as non-responders in the analysis. Regarding the follow-up duration, while some migraine education studies have followed patients for 12 months [57], we opted for a shorter follow-up period, as in other trials [58], in order to grant a good preventive prediction of neurophysiological tests. Longer follow-ups could be influenced by spontaneous fluctuations that may alter the headache profile [59]. In conclusion, despite the complexities of the TNE protocol, the study incorporates strategies to minimize dropouts. The increasing use of TNE in challenging settings, such as for breast cancer patients [60], demonstrates the high feasibility of these approaches.

## Data Availability

No new data were created or analyzed in this study. Data sharing is not applicable to this article.
